# Skin Grafting and Rhinoplasty

**Published:** 1884-09

**Authors:** 


SKIN GRAFTING AND RHINOPLASTY.—Thomas Colt has recently been discharged from Bellevue Hospital with a restored nose. He was deprived of his nose a number of years ago by a cancerous affection technically called lupus, which destroyed the nasal bone as well as the fleshy covering, and even the lower eyelids. His treatment was undertaken over ten years ago by Dr. Thomas Sabine, the Professor of Anatomy of the College of Physicians and Surgeons, and has been successfully pursued up to the present time. Dr. Sabine first addressed himself to the task of arresting the disease, and when that was accomplished he restored the lost eyelids by grafting thereon healthy skin taken from the cheeks and forehead of the patient. The more difficult operation of restoring the nose followed. This was done by making use of the third finger of the left hand, from which the nail was first removed by nitric acid. Then the end of the finger was fixed against the forehead between the eyes, the epidermis at the points of contact having been previously removed to bring about adhesion. At the same time the finger up to the second joint was split open on the under side, the flesh stripped off, and the flaps thereby produced were connected with the flesh of the cheek on either side. The hand was fixed in the proper position by plaster of Paris and held so until the adhesion was com-complete. Then the finger was amputated at the second joint, and the free edges of the part adhering to the face were arranged so as to form the wings of the nostrils. During all this time the nasal orifice was kept open by a hard rubber tube. The treatment necessarily occupied much time, and involved a number of painful operations, but was completely successful, and it is almost impossible now to distinguish the nose thus fashioned by surgical skill from one cast in nature's own mold.

